# Development and validation of a predictive nomogram for osteoporosis in patients with psoriasis

**DOI:** 10.3389/fmed.2026.1826823

**Published:** 2026-07-10

**Authors:** Zhilong Wang, Zhiyou Zhou, Ruichen Jiang, Haoning Ma, Yanzhu Shen, Xiangsheng Tang, Ping Yi, Yi Li, Zihan Wei

**Affiliations:** 1 Dongzhimen Hospital, Beijing University of Chinese Medicine, Beijing, China; 2 Department of Orthopedics, Third Affiliated Hospital of Naval Medical University, Shanghai, China; 3 Department of Clinical Medicine, Beijing University of Chinese Medicine, Beijing, China; 4 Department of Orthopaedics, China-Japan Friendship Hospital, Beijing, China; 5 Beijing Hospital, National Centre of Gerontology, Beijing, China

**Keywords:** nomogram, osteoporosis, psoriasis, psoriatic arthritis, risk prediction, vitamin D

## Abstract

**Background:**

Psoriasis is a chronic inflammatory disease associated with increased risk of osteoporosis. However, practical tools for identifying high-risk patients in clinical settings remain limited. This study aimed to develop and validate a nomogram for predicting osteoporosis in patients with psoriasis.

**Methods:**

A retrospective cohort study was conducted including 639 psoriasis patients from January 2023 to October 2025. Patients were randomly divided into training (*n* = 447) and validation (*n* = 192) sets at a 7:3 ratio. Osteoporosis was defined as dual-energy X-ray absorptiometry (DXA) *T*-score ≤−2.5. Univariate analysis and multivariate logistic regression were performed to identify independent risk factors. A nomogram was constructed based on the final model and evaluated using C-index, calibration curves, and decision curve analysis (DCA).

**Results:**

The overall prevalence of osteoporosis was 11.7% (75/639). Six independent risk factors were identified: advanced age (OR = 4.054, 95%CI 2.281–7.237), disease duration (OR = 1.068, 95%CI 1.026–1.11), psoriatic arthritis (OR = 2.178, 95%CI 1.243–3.796), vitamin D deficiency (OR = 5.148, 95%CI 1.986–17.672), systemic corticosteroid use (OR = 3.501, 95%CI 1.756–6.826), male sex (OR = 0.399, 95%CI 0.229–0.686). The nomogram demonstrated good discrimination with AUC of 0.824 in the training set and 0.771 in the validation set. Calibration curves showed excellent agreement between predicted and observed probabilities. DCA confirmed the clinical utility of the model.

**Conclusion:**

We developed and validated a practical nomogram incorporating psoriasis-specific and traditional risk factors for predicting osteoporosis risk. This tool may facilitate early identification of high-risk patients and guide clinical decision-making for DXA screening and preventive interventions.

## 1 Introduction

Psoriasis is a chronic, immune-mediated inflammatory disease driven by dysregulated innate and adaptive immunity, characterized by elevated levels of pro-inflammatory cytokines including tumor necrosis factor-alpha (TNF-*α*), interleukin-17 (IL-17), and interleukin-23 (IL-23), and may involve systemic manifestations beyond the skin (1). Psoriatic arthritis (PsA), a major subtype affecting up to 30% of psoriasis patients, often indicates higher inflammatory burden and more complex comorbidity profiles (2).

Osteoporosis, characterized by reduced bone mineral density (BMD) and deteriorated bone microarchitecture, is a major cause of fragility fractures with high morbidity, mortality, and healthcare costs (3). The gold standard diagnostic criterion is dual-energy X-ray absorptiometry (DXA) with T-score ≤ − 2.5 (4). However, DXA is not universally accessible in all healthcare settings, and bone health risk is not solely determined by BMD thresholds, highlighting the need for practical risk stratification strategies in specific patient populations (5).

From a pathophysiological perspective, there is strong biological plausibility for increased osteoporosis risk in psoriasis patients. Chronic systemic inflammation may alter bone remodeling by disrupting the balance between osteoblast and osteoclast activity (6). Additionally, PsA-related pain and reduced mobility may accelerate bone loss, while glucocorticoid exposure represents an additive risk factor that can rapidly induce bone loss and significantly increase fracture risk (7, 8). Furthermore, multiple systematic reviews and meta-analyses have documented lower vitamin D levels in psoriasis patients, with potential associations with bone metabolism markers such as parathyroid hormone (PTH), supporting the inclusion of 25-hydroxyvitamin D [25(OH)D] in bone health risk assessment (9, 10).

Current evidence regarding the association between psoriasis and the risk of osteoporosis and fractures remains somewhat inconsistent, with observed heterogeneity attributable to differences in disease subtype, severity, treatment modalities, and lifestyle factors. Nevertheless, systematic reviews and meta-analyses have consistently indicated an elevated risk of osteoporosis in patients with psoriasis and psoriatic arthritis (11). In particular, patients with comorbid PsA, longer disease duration, greater inflammatory burden, and a history of glucocorticoid exposure represent a clinically identifiable high-risk population that may benefit from targeted bone health screening. This heterogeneity in the literature does not negate the clinical necessity of developing disease-specific risk stratification tools; on the contrary, it underscores the importance of incorporating psoriasis-specific variables that are overlooked by general population assessment tools. At present, bone health management has not been adequately integrated into routine comorbidity screening workflows for psoriasis, representing an important clinical gap.

In the general population, tools such as the Fracture Risk Assessment Tool (FRAX) integrate clinical risk factors including age, sex, body mass index (BMI), prior fragility fracture, parental hip fracture, smoking, long-term oral glucocorticoid use, and excessive alcohol consumption to estimate future fracture probability (12). However, FRAX does not explicitly incorporate disease severity (e.g., Psoriasis Area and Severity Index [PASI]), PsA status, inflammatory burden (C-reactive protein [CRP]/erythrocyte sedimentation rate [ESR]), or vitamin D levels—potentially critical determinants in psoriasis patients. Moreover, the complex treatment landscape and comorbidity structure in psoriasis may introduce significant confounding and indication bias, creating uncertainty regarding the discrimination and calibration performance of general population tools in this context.

Based on this background, we aimed to develop and validate a predictive nomogram specifically for psoriasis patients to identify those at high risk for osteoporosis. This model integrates traditional osteoporosis risk factors with psoriasis-specific information and inflammatory/metabolic markers, providing a quantitative tool more aligned with the clinical context of psoriasis for guiding DXA screening decisions and fracture prevention interventions.

## 2 Materials and methods

### 2.1 Study design

This retrospective cohort study was conducted in strict accordance with the Transparent Reporting of a multivariable prediction model for Individual Prognosis Or Diagnosis (TRIPOD) guidelines (13) and stepwise guidelines for clinical prediction model development (14).

### 2.2 Ethics approval

This study was approved by the Ethics Committee of Dazhou integrated TCM & Western Medicine Hospital (Approval No.: 2025106). Given the retrospective nature of the study and complete anonymization of all research data, the ethics committee waived the requirement for individual informed consent.

### 2.3 Study population

The study population comprised patients with psoriasis who presented to the Department of Dermatology at our hospital between January 1, 2023, and October 31, 2025. Inclusion criteria:

Confirmed diagnosis of psoriasis based on clinical and/or histological criteria; Age ≥18 years. Exclusion criteria: Missing clinical data >20%; Concurrent severe immune-related diseases (e.g., leukemia, nephrotic syndrome, cirrhosis, acquired immunodeficiency syndrome).

### 2.4 Sample size calculation

Sample size was calculated based on the “events per variable” (EPV) principle for clinical prediction models, requiring at least 10 outcome events per predictor variable (16). Based on prior literature, we anticipated 5–7 predictor variables and an osteoporosis prevalence of 12–18%. Conservatively assuming 7 variables and 12% prevalence, the minimum required sample size was 584 patients (7 variables × 10 EPV/0.12 = 583.3). Accounting for data quality and missingness, we ultimately enrolled 639 patients with 75 osteoporosis cases (11.7%), satisfying the EPV requirement for the final 6-variable model.

### 2.5 Outcome definition

The primary endpoint was the presence of osteoporosis, defined as DXA T-score ≤ − 2.5 at the lumbar spine, femoral neck, or total hip.

### 2.6 Data collection

Variable collection followed a systematic approach. We first conducted a comprehensive literature review to identify risk factors associated with osteoporosis in psoriasis patients, drawing on established associations documented in prior studies on osteoporosis and psoriasis/PsA (11, 15, 16). Combined with clinical expertise, a total of 26 candidate variables were selected for analysis (Table 1).

**Table 1:** Baseline characteristics of training and validation sets.

Characteristics	Training	Validation	*t/z/χ^2^*	*p*
Age, years			0.76	0.383
≤60	356 (79.64)	147 (76.56)		
>60	91 (20.36)	45 (23.44)		
Sex			0.809	0.368
Female	190 (42.51)	89 (46.35)		
Male	257 (57.49)	103 (53.65)		
Smoking status			0.001	0.972
No	344 (76.96)	148 (77.08)		
Yes	103 (23.04)	44 (22.92)		
Alcohol consumption			1.016	0.314
No	398 (89.04)	176 (91.67)		
Yes	49 (10.96)	16 (8.33)		
Physical activity level			0.456	0.796
High	86 (19.24)	34 (17.71)		
Low	156 (34.9)	72 (37.5)		
Mid	205 (45.86)	86 (44.79)		
Psoriatic arthritis			1.481	0.224
No	335 (74.94)	135 (70.31)		
Yes	112 (25.06)	57 (29.69)		
Serum 25-hydroxyvitamin D, ng/mL			0.163	0.687
≤30	90 (20.13)	36 (18.75)		
>30	357 (79.87)	156 (81.25)		
Type 2 diabetes mellitus			0.921	0.337
No	358 (80.09)	160 (83.33)		
Yes	89 (19.91)	32 (16.67)		
Hypertension			0.102	0.749
No	264 (59.06)	116 (60.42)		
Yes	183 (40.94)	76 (39.58)		
Dyslipidemia			0.028	0.867
No	309 (69.13)	134 (69.79)		
Yes	138 (30.87)	58 (30.21)		
Systemic corticosteroid use			0.312	0.576
No	401 (89.71)	175 (91.15)		
Yes	46 (10.29)	17 (8.85)		
Methotrexate use			0.003	0.956
No	332 (74.27)	143 (74.48)		
Yes	115 (25.73)	49 (25.52)		
Cyclosporine use			0.022	0.882
No	393 (87.92)	168 (87.5)		
Yes	54 (12.08)	24 (12.5)		
Biologic agent use			0.115	0.734
No	244 (54.59)	102 (53.12)		
Yes	203 (45.41)	90 (46.88)		
Osteoporosis			0.169	0.681
No	393 (87.92)	171 (89.06)		
Yes	54 (12.08)	21 (10.94)		
Body mass index, kg/m^2^	25.1 ± 3.85	25.18 ± 3.92	0.227	0.82
Daily sun exposure, hours/week	5.3 (3.7, 7.1)	5 (2.98, 6.9)	−1.711	0.087
Disease duration, years	7.4 (4.4, 10.25)	7.4 (3.95, 10)	−0.667	0.505
Psoriasis Area and Severity Index	8 (5.2, 13.25)	8.35 (5.47, 12.98)	0.642	0.521
Body surface area involvement, %	10.6 (4.9, 15.9)	10.55 (5.9, 15.32)	0.212	0.832
Dermatology Life Quality Index	8 (4, 11)	8 (5, 11)	0.37	0.711
C-reactive protein, mg/L	4.34 (2.99, 6.64)	4.75 (3.06, 6.99)	1.097	0.273
Erythrocyte sedimentation rate, mm/h	15.2 (11.1, 21.9)	15.15 (10.3, 22.13)	0.068	0.946
Serum calcium, mmol/L	2.28 (2.24, 2.34)	2.28 (2.23, 2.34)	−0.337	0.736
Serum phosphate, mmol/L	1.09 ± 0.15	1.08 ± 0.14	−0.219	0.826
Alkaline phosphatase, U/L	86 (69, 102.5)	87 (70, 103.25)	0.663	0.507
Parathyroid hormone, pg/mL	45.49 ± 12.52	45.67 ± 11.33	0.168	0.866

Data were retrospectively extracted from the hospital electronic medical record (EMR) system by two research team members using a standardized Excel template. All data underwent dual verification and cross-checking by the two extractors, with random auditing by a third team member to ensure accuracy and reliability.

Missing data handling: Patients with missing clinical data >20% were excluded per the exclusion criteria. For retained variables, complete case analysis was applied, as the proportion of missing data for all retained variables was low (<5%). Sensitivity analyses confirmed that results were consistent with and without imputation.

### 2.7 Statistical analysis

Continuous variables were reported as median (interquartile range [IQR]) or mean ± standard deviation (SD) based on distribution characteristics. Categorical variables were presented as frequencies (percentages). Chi-square tests were used to compare categorical variables between groups, and Mann–Whitney *U* tests were used for continuous variables. Univariate analysis and multivariate logistic regression were performed on candidate variables. Variables with *p* < 0.05 in both analyses were identified as independent risk factors for osteoporosis in psoriasis patients. Independent risk factors were incorporated into a nomogram using the “rms” package to construct a dynamic predictive model. Model discrimination was assessed using the area under the receiver operating characteristic curve (AUC). Calibration was evaluated using calibration curves to assess the agreement between predicted and observed probabilities. Clinical utility was quantified using decision curve analysis (DCA), plotting net benefit across different threshold probabilities. All statistical analyses were performed using R 4.2.0. Two-sided *p*-values <0.05 were considered statistically significant.

Regarding the variable selection strategy: a two-stage approach (univariate screening followed by multivariate analysis) was adopted rather than LASSO regression or AIC-based stepwise selection, for the following reasons: (1) the number of candidate variables was relatively limited (*n* = 26) and the number of outcome events was modest (*n* = 75), under which conditions LASSO regression may exhibit instability; (2) clinical interpretability was prioritized, and the two-stage approach is consistent with commonly used methodological practices in clinical prediction model development; (3) although univariate screening may increase the risk of type I error due to multiple comparisons, the subsequent multivariate step provided additional variable filtering.

## 3 Results

### 3.1 Baseline characteristics and balance analysis

A total of 639 psoriasis patients meeting inclusion criteria were enrolled and randomly divided into training (*n* = 447, 70%) and validation (*n* = 192, 30%) sets. The overall prevalence of osteoporosis was 11.7% (75/639), with 54 cases (12.1%) in the training set and 21 cases (10.9%) in the validation set (*p* = 0.681). Baseline characteristics showed no significant differences between the two sets for all variables (all *p* > 0.05), confirming successful randomization (Table 1).

### 3.2 Univariate analysis

Univariate analysis revealed significant associations (*p* < 0.05) between osteoporosis and the following variables: advanced age (≥50 years: 46.67% vs. 17.91%, *p* < 0.001), female sex (56.0% vs. 42.02%, *p* = 0.022), disease duration (median 10.7 vs. 7.3 years, p < 0.001), PsA (45.33% vs. 23.94%, *p* < 0.001), vitamin D deficiency (94.67% vs. 78.37%, *p* = 0.001), hypertension (53.33% vs. 38.83%, *p* = 0.016), and systemic corticosteroid use (25.33% vs. 7.80%, *p* < 0.001) (Table 2).

**Table 2:** Univariate analysis of factors associated with osteoporosis.

Characteristics	Without osteoporosis	With osteoporosis	*t/z/χ^2^*	*p*
Body mass index, kg/m^2^	25.19 ± 3.95	24.63 ± 3.2	1.383	0.17
Daily sun exposure, hours/week	5.2 (3.4, 7)	5.4 (4.1, 7.4)	−1.444	0.149
Disease duration, years	7.3 (4.27, 9.6)	10.7 (9.2, 13.75)	−5.716	<0.001
Psoriasis Area and Severity Index	8 (5.18, 12.72)	9.3 (5.95, 15.45)	−1.76	0.078
Body surface area involvement, %	10.3 (4.8, 15.2)	11.8 (6.7, 18.05)	−2.114	0.035
Dermatology Life Quality Index	8 (4, 11)	8 (5, 11.5)	−1.292	0.195
C-reactive protein, mg/L	4.36 (2.97, 6.79)	4.79 (3.47, 7.19)	−1.22	0.223
Erythrocyte sedimentation rate, mm/h	15.1 (10.7, 21.9)	16.9 (11.65, 22.7)	−1.065	0.287
Serum calcium, mmol/L	2.28 (2.23, 2.34)	2.29 (2.21, 2.34)	0.042	0.967
Serum phosphate, mmol/L	1.08 (0.99, 1.17)	1.08 (1.02, 1.2)	−0.784	0.433
Alkaline phosphatase, U/L	86 (69, 104)	82 (69, 100.5)	0.647	0.518
Parathyroid hormone, pg/mL	45.55 ± 12.24	45.54 ± 11.64	0.007	0.995
Age, years			32.68	<0.001
≤60	463 (82.09)	40 (53.33)		
>60	101 (17.91)	35 (46.67)		
Sex			5.259	0.022
Female	237 (42.02)	42 (56)		
Male	327 (57.98)	33 (44)		
Smoking status			0.048	0.827
No	435 (77.13)	57 (76)		
Yes	129 (22.87)	18 (24)		
Alcohol consumption			3.158	0.076
No	511 (90.6)	63 (84)		
Yes	53 (9.4)	12 (16)		
Physical activity level			5.662	0.059
High	108 (19.15)	12 (16)		
Low	192 (34.04)	36 (48)		
Mid	264 (46.81)	27 (36)		
Psoriatic arthritis			15.58	<0.001
No	429 (76.06)	41 (54.67)		
Yes	135 (23.94)	34 (45.33)		
Serum 25-hydroxyvitamin D, ng/mL			11.107	0.001
≤30	122 (21.63)	4 (5.33)		
>30	442 (78.37)	71 (94.67)		
Type 2 diabetes mellitus			0.142	0.706
No	456 (80.85)	62 (82.67)		
Yes	108 (19.15)	13 (17.33)		
Hypertension			5.777	0.016
No	345 (61.17)	35 (46.67)		
Yes	219 (38.83)	40 (53.33)		
Dyslipidemia			3.476	0.062
No	398 (70.57)	45 (60)		
Yes	166 (29.43)	30 (40)		
Systemic corticosteroid use			22.895	<0.001
No	520 (92.2)	56 (74.67)		
Yes	44 (7.8)	19 (25.33)		
Methotrexate use			0.4	0.527
No	417 (73.94)	58 (77.33)		
Yes	147 (26.06)	17 (22.67)		
Cyclosporine use			1.141	0.285
No	498 (88.3)	63 (84)		
Yes	66 (11.7)	12 (16)		
Biologic agent use			0.023	0.88
No	306 (54.26)	40 (53.33)		
Yes	258 (45.74)	35 (46.67)		

### 3.3 Multivariate logistic regression

Multivariate logistic regression identified six independent risk factors for osteoporosis: (1) age ≥50 years (OR = 4.054, 95%CI 2.281–7.237, *p* < 0.001); (2) disease duration (OR = 1.068 per year, 95%CI 1.026–1.11, *p* = 0.001); (3) PsA (OR = 2.178, 95%CI 1.243–3.796, *p* = 0.006); (4) vitamin D deficiency (OR = 5.148, 95%CI 1.986–17.672, *p* = 0.003); (5) systemic corticosteroid use (OR = 3.501, 95%CI 1.756–6.826, p < 0.001); (6) male sex as protective (OR = 0.399, 95%CI 0.229–0.686, *p* = 0.001) (Table 3).

**Table 3:** Multivariate logistic regression analysis of independent risk factors for osteoporosis.

Characteristics	Estimate	Std. error	Statistic	*p*	OR (95%CI)
Disease duration, years	0.065	0.020	3.249	0.001	1.068(1.026 ~ 1.11)
Body surface area involvement, %	0.015	0.017	0.919	0.358	1.015(0.982 ~ 1.049)
Age, years					
≤60	R				
>60	1.4	0.294	4.767	<0.001	4.054(2.281 ~ 7.237)
Sex					
Female	R				
Male	−0.918	0.279	−3.287	0.001	0.399(0.229 ~ 0.686)
Psoriatic arthritis					
No	R				
Yes	0.778	0.284	2.743	0.006	2.178(1.243 ~ 3.796)
Serum 25-hydroxyvitamin D, ng/mL					
≤30	R				
>30	1.639	0.544	3.012	0.003	5.148(1.986 ~ 17.672)
Systemic corticosteroid use					
No	R				
Yes	1.253	0.345	3.634	<0.001	3.501(1.756 ~ 6.826)

### 3.4 Nomogram development

**Figure 1:**
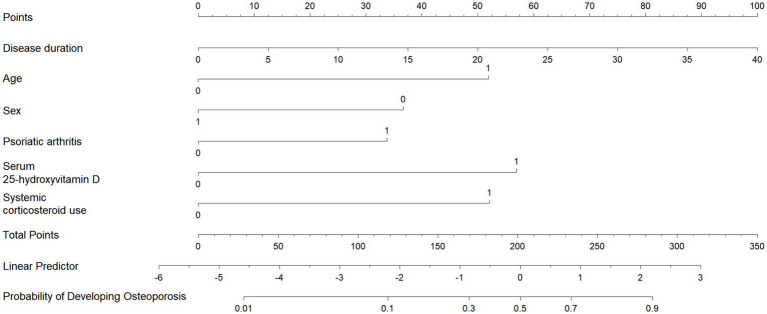
Nomogram for predicting osteoporosis risk in patients with psoriasis.

Based on the six independent risk factors identified in multivariate analysis, we constructed a nomogram for predicting osteoporosis risk in psoriasis patients (Figure 1). The nomogram assigns point values to each predictor, with total points corresponding to individualized osteoporosis probability. This visual tool enables rapid risk assessment at the point of care.

### 3.5 Model performance evaluation

#### 3.5.1 Discrimination

**Figure 2:**
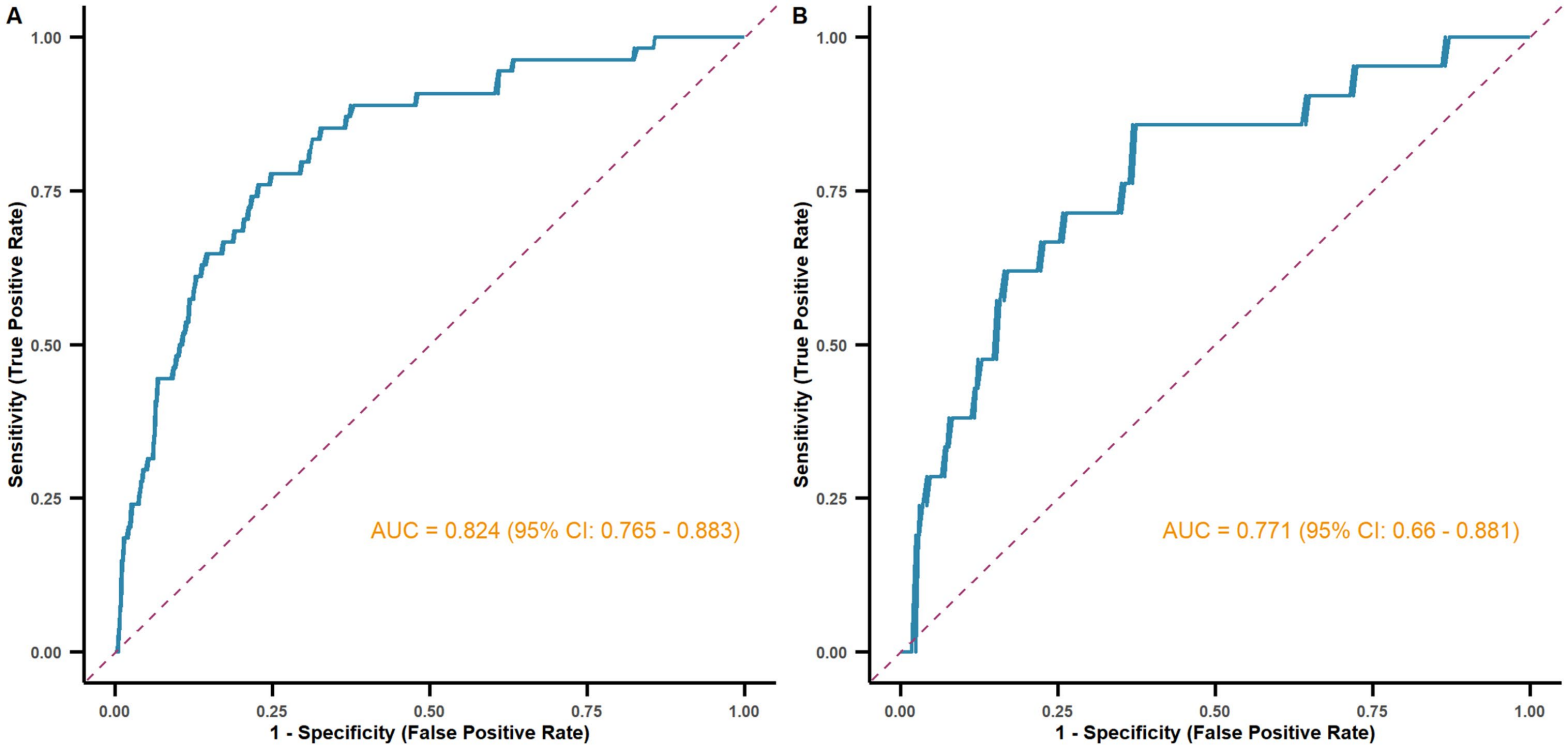
Receiver operating characteristic (ROC) curves for the nomogram. **(A)** Training set; **(B)** validation set.

The nomogram demonstrated excellent discrimination ability. ROC analysis showed AUC of 0.824 for the training set and 0.771 for the validation set, confirming robust predictive accuracy (Figure 2).

#### 3.5.2 Calibration

**Figure 3:**
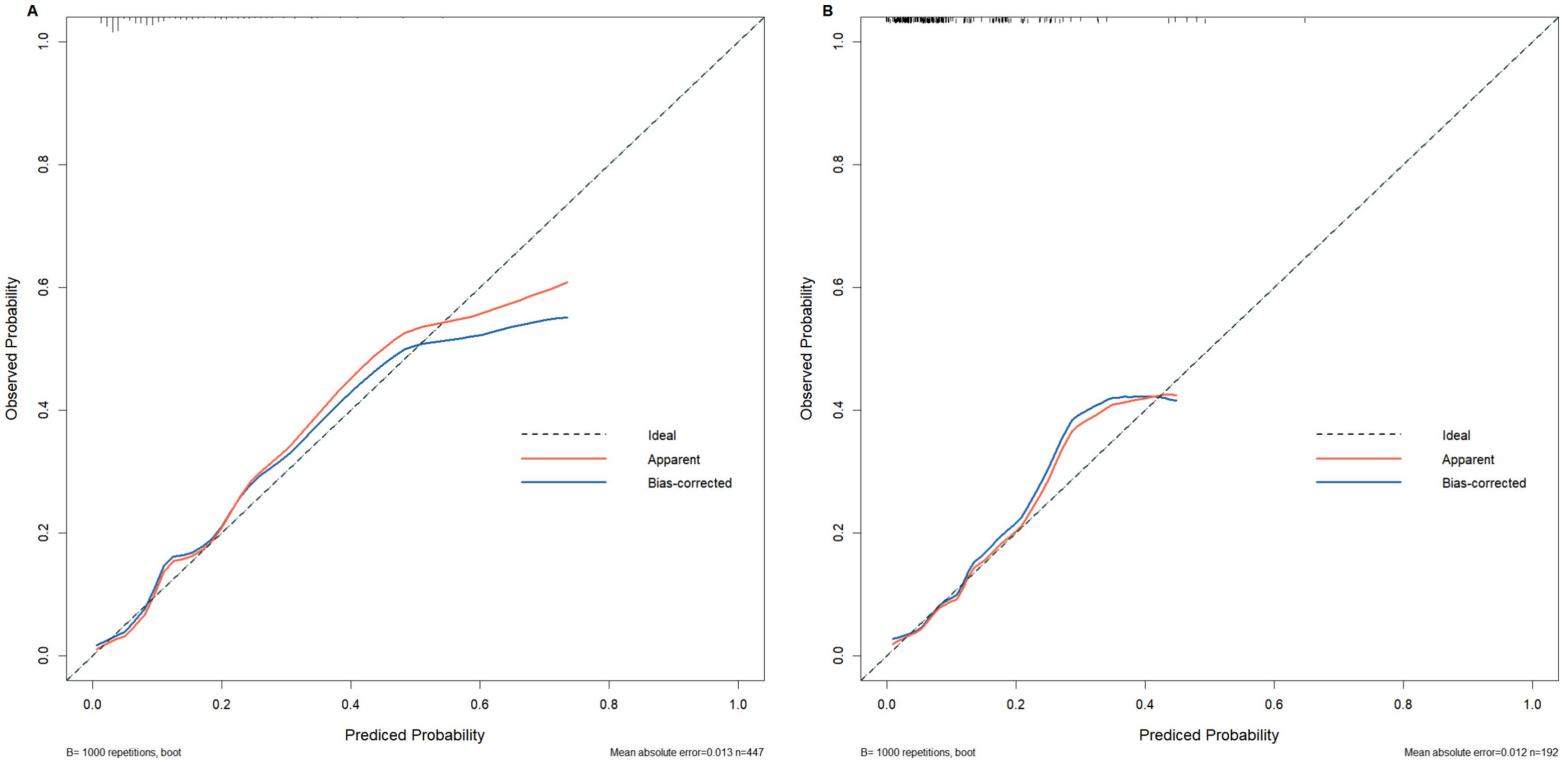
Calibration curves for the nomogram. **(A)** Training set; **(B)** validation set.

Calibration curves demonstrated excellent agreement between predicted probabilities and observed outcomes in both training and validation sets (Figure 3). The calibration slope approached 1.0 with minimal deviation from the ideal 45-degree reference line, indicating accurate probability estimates across the risk spectrum. However, the predicted probability range in the validation set calibration curve (Figure 3B) was approximately 0–0.45, reflecting the relatively low prevalence of osteoporosis in the present sample. Calibration performance beyond this range could not be directly assessed in the current cohort, and future validation in populations with higher osteoporosis prevalence is warranted.

#### 3.5.3 Clinical utility

**Figure 4:**
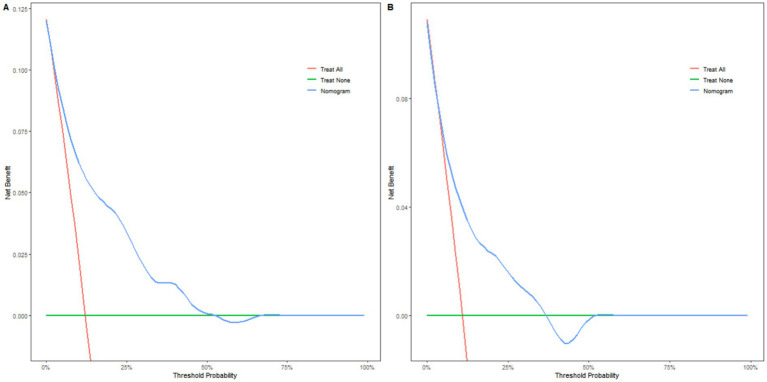
Decision curve analysis (DCA) for the nomogram. **(A)** Training set; **(B)** validation set.

Decision curve analysis confirmed the clinical utility of the nomogram (Figure 4). The model provided positive net benefit across a wide range of threshold probabilities (5–60%), substantially outperforming both “treat all” and “treat none” strategies. This indicates that the nomogram can effectively guide clinical decisions regarding DXA screening and preventive interventions.

## 4 Discussion

To our knowledge, this is the first study to develop and validate a comprehensive nomogram specifically designed to predict osteoporosis risk in psoriasis patients. By integrating traditional osteoporosis risk factors with psoriasis-specific clinical features and biomarkers, our model achieved excellent discrimination, calibration, and clinical utility. This tool addresses an important clinical gap, as bone health screening is not routinely incorporated into psoriasis comorbidity management protocols despite mounting evidence of increased fracture risk in this population.

Age emerged as the strongest predictor of osteoporosis in our cohort. This finding aligns with established age-related bone loss mechanisms, wherein the balance between bone formation and resorption shifts progressively toward resorption. Chandran et al. (15) demonstrated that age correlates inversely with BMD in PsA patients, while Chen et al. (11) identified age as a key moderator in BMD analyses, with patients ≥50 years showing more pronounced lumbar spine BMD changes. The accelerated bone loss observed in older psoriasis patients likely reflects synergistic effects of aging and chronic inflammation. Moosazadeh et al. (16) meta-analyzed 23 studies comprising 1,876 psoriasis patients and 7,532 controls, revealing significantly lower serum 25(OH)D levels in psoriasis patients (21.0 ± 8.3 vs. 28.8 ± 10.3 ng/mL, *p* < 0.001) along with elevated PTH (43.7 ± 16.5 vs. 38.7 ± 12.8 pg/mL, *p* = 0.015). The PTH elevation likely represents a compensatory response to maintain calcium homeostasis in the setting of vitamin D insufficiency. Multiple mechanisms may contribute to vitamin D deficiency in psoriasis: reduced sun exposure due to covering skin lesions for cosmetic reasons, and lower expression of CYP27A1 and CYP27B1 enzymes in psoriatic keratinocytes, which impairs cutaneous vitamin D synthesis (8, 17). Given the essential role of vitamin D in calcium-phosphate homeostasis and bone mineralization (18), our findings support routine vitamin D assessment and supplementation as part of comprehensive psoriasis management. Johannesdottir et al. (19) analyzed 723,251 Danish adults and found dose- and duration-dependent associations between topical corticosteroid use and both osteoporosis (high-dose HR = 1.27, 95%CI 1.16–1.39) and major osteoporotic fractures (high-dose HR = 1.12, 95%CI 1.02–1.23). Glucocorticoids impair bone health through multiple pathways: suppressing osteoblast differentiation, enhancing osteoclast activity, reducing intestinal calcium absorption, and interfering with vitamin D metabolism (20). These findings emphasize the need for bone-protective strategies in psoriasis patients requiring prolonged Kocijan et al. (21) employed high-resolution peripheral quantitative computed tomography to demonstrate significant reductions in trabecular BMD (−12.0%, *p* = 0.021), bone volume fraction (−11.9%, *p* = 0.020), and trabecular number (−7.1%, *p* = 0.035) at the distal radius in PsA patients versus controls. Borman et al. (22) reported negative correlations between arthritis duration and both lumbar spine (*r* = −0.48, *p* < 0.05) and total hip (*r* = −0.52, *p* < 0.05) BMD values. The heightened osteoporosis risk in PsA likely reflects local inflammatory microenvironments at affected joints, reduced physical activity secondary to joint dysfunction and pain, and excessive osteoclast activation driven by inflammatory cytokines including TNF-*α* and RANKL (23). D’Epiro et al. (24) found that psoriasis patients with osteopenia/osteoporosis had significantly longer disease duration (17.0 ± 9.3 years) compared to those with normal BMD (8.8 ± 6.1 years, *p* = 0.04). These findings suggest that cumulative inflammatory burden drives progressive bone loss, supporting the concept of subclinical musculoskeletal involvement even in patients without overt arthritis. Sex differences revealed an unexpected pattern, with male sex appearing protective (OR = 0.399, *p* = 0.001). This contrasts with general population data showing higher osteoporosis prevalence in women, particularly post-menopausal women due to estrogen deficiency (25). However, Dreiher et al. (26) reported a sex-specific association in their Israeli case–control study (7,936 psoriasis cases, 14,835 controls), finding significantly elevated osteoporosis prevalence in male psoriasis patients versus male controls (3.1% vs. 1.7%, OR = 1.86, *p* < 0.001), but no significant difference among women. This suggests that psoriasis may disproportionately increase male osteoporosis risk, although the underlying mechanisms remain unclear and warrant further investigation (27). Our finding may reflect our study population’s age and sex distribution or indicate that inflammation differentially affects bone metabolism across sexes.

Our nomogram offers several advantages for clinical practice. First, it incorporates readily available clinical and laboratory parameters, enabling risk assessment without additional diagnostic procedures. Second, the visual format facilitates rapid calculation at the point of care. Third, the model demonstrated robust performance in internal validation, with consistent discrimination and calibration. Fourth, decision curve analysis confirmed clinically meaningful net benefit across relevant threshold probabilities, supporting its use to guide DXA screening decisions. Specifically, the nomogram can help identify high-risk patients who would most benefit from early DXA evaluation and preventive interventions including vitamin D supplementation, calcium intake optimization, weight-bearing exercise, smoking cessation, and consideration of anti-osteoporotic pharmacotherapy. These findings are consistent with those of prior studies (28, 29).

This study has several strengths, including adherence to TRIPOD guidelines, comprehensive assessment of psoriasis-specific and traditional risk factors, rigorous statistical methodology with internal validation, and evaluation of clinical utility via decision curve analysis. However, important limitations must be acknowledged. First, the retrospective single-center design may limit the generalizability of our findings. Second, only internal validation was performed, and prospective multicenter studies are needed to confirm the model’s external validity. Third, residual confounding from unmeasured variables cannot be excluded. Fourth, bone microarchitectural indices beyond DXA were not assessed. Fifth, the cross-sectional outcome ascertainment precluded evaluation of incident fracture risk. Sixth, the calibration curve in the validation cohort showed a predicted probability range truncated at approximately 0.45, which constrains our ability to evaluate calibration performance in higher-risk patients; this likely reflects the relatively low prevalence of osteoporosis in the present cohort (11.7%), and future studies should validate the model in populations with higher disease prevalence. Seventh, systemic corticosteroid use was treated as a binary variable rather than incorporating cumulative dose, representing a pragmatic simplification; future research should quantify cumulative glucocorticoid exposure to better characterize this risk. Eighth, the two-stage univariate-to-multivariate screening strategy for candidate variable selection may be associated with an elevated type I error rate during the screening phase. Ninth, subgroup analyses by PsA status or biologic agent class (e.g., anti-TNF versus IL-17 inhibitors) were not feasible given the current sample size, and larger studies are needed to explore potential effect modification across these subgroups.

Future research should focus on external validation in diverse populations, prospective studies evaluating fracture outcomes, assessment of whether nomogram-guided interventions improve patient outcomes, incorporation of additional biomarkers (e.g., bone turnover markers, inflammatory cytokines), and development of web-based or mobile applications to enhance clinical accessibility. Additionally, investigating whether biologic therapies targeting key inflammatory pathways (IL-17, IL-23, TNF-α) may modify osteoporosis risk would be of considerable interest.

## 5 Conclusion

We developed and internally validated a practical nomogram incorporating six independent risk factors—age, disease duration, psoriatic arthritis, vitamin D deficiency, systemic corticosteroid use, and sex—to predict osteoporosis risk in psoriasis patients. The model demonstrated excellent discrimination, calibration, and clinical utility, offering a quantitative tool to guide DXA screening decisions and preventive interventions. By integrating psoriasis-specific features with traditional osteoporosis risk factors, this nomogram addresses an important gap in psoriasis comorbidity management. External validation and prospective evaluation are warranted to confirm its generalizability and clinical impact.

## Data Availability

The original contributions presented in the study are included in the article/supplementary material, further inquiries can be directed to the corresponding authors.
